# Predictors of maternal health services utilization by poor, rural women: a comparative study in Indian States of Gujarat and Tamil Nadu

**DOI:** 10.1186/s41043-015-0025-x

**Published:** 2015-07-31

**Authors:** Kranti Suresh Vora, Sally A. Koblinsky, Marge A. Koblinsky

**Affiliations:** 1 Indian Institute of Public Health Gandhinagar, Drive-in-Road, Ahmedabad, Gujarat 380054 India; 2 University of Maryland, College Park,, Prince George’s; 3 USAID, Washington D.C, USA

**Keywords:** Maternal health services, Poor rural women, India, Cash transfer schemes

## Abstract

**Background:**

India leads all nations in numbers of maternal deaths, with poor, rural women contributing disproportionately to the high maternal mortality ratio. In 2005, India launched the world’s largest conditional cash transfer scheme, *Janani Suraksha Yojana* (JSY), to increase poor women’s access to institutional delivery, anticipating that facility-based birthing would decrease deaths. Indian states have taken different approaches to implementing JSY. Tamil Nadu adopted JSY with a reorganization of its public health system, and Gujarat augmented JSY with the state-funded *Chiranjeevi Yojana* (CY) scheme, contracting with private physicians for delivery services. Given scarce evidence of the outcomes of these approaches, especially in states with more optimal health indicators, this cross-sectional study examined the role of JSY/CY and other healthcare system and social factors in predicting poor, rural women’s use of maternal health services in Gujarat and Tamil Nadu.

**Methods:**

Using the District Level Household Survey (DLHS)-3, the sample included 1584 Gujarati and 601 Tamil rural women in the lowest two wealth quintiles. Multivariate logistic regression analyses examined associations between JSY/CY and other salient health system, socio-demographic, and obstetric factors with three outcomes: adequate antenatal care, institutional delivery, and Cesarean-section.

**Results:**

Tamil women reported greater use of maternal healthcare services than Gujarati women. JSY/CY participation predicted institutional delivery in Gujarat (AOR = 3.9), but JSY assistance failed to predict institutional delivery in Tamil Nadu, where mothers received some cash for home births under another scheme. JSY/CY assistance failed to predict adequate antenatal care, which was not incentivized. All-weather road access predicted institutional delivery in both Tamil Nadu (AOR = 3.4) and Gujarat (AOR = 1.4). Women’s education predicted institutional delivery and Cesarean-section in Tamil Nadu, while husbands’ education predicted institutional delivery in Gujarat.

**Conclusions:**

Overall, assistance from health financing schemes, good road access to health facilities, and socio-demographic and obstetric factors were associated with differential use of maternity health services by poor, rural women in the two states. Policymakers and practitioners should promote financing schemes to increase access, including consideration of incentives for antenatal care, and address health system and social factors in designing state-level interventions to promote safe motherhood.

## Background

India has implemented national and state-level strategies and programs to improve women’s use of maternal health services, including Child Survival Safe Motherhood (CSSM) and Reproductive Child Health I and II. With a focus on supply-side interventions, these programs enhanced health infrastructure, skilled personnel availability, referral linkages, and blood transfusion accessibility during childbirth [1]. Yet, India continues to lead globally in numbers of maternal deaths, with poor women in rural areas at greatest risk for maternal mortality [2, 3]. Limited uptake of maternity health services among this group of women threatens the country’s ability to reach Millennium Development Goals four and five [3].

Under the National Rural Health Mission (NRHM), India introduced schemes to increase demand for maternal health care [1]. In 2005–06, the nation launched the world’s largest conditional cash transfer scheme for maternal health, *Janani Suraksha Yojana* (JSY), or a “scheme to protect mothers.” JSY aims to reduce maternal and neonatal deaths by providing cash incentives to mothers who give birth in a health institution [1, 3]. The overall framework for JSY is similar throughout India, but states have different eligibility criteria and incentives based on their institutional birth data when the scheme was created [4]. In states with high maternal mortality and low institutional deliveries (low performing states), women receive cash incentives for institutional births in public or accredited health facilities regardless of socioeconomic status, age, or parity. In states with better maternal and institutional delivery data (high performing states), cash incentives are limited to the first two live births of women below the poverty line (BPL) and from scheduled castes and tribes [4].

Another maternal healthcare financing scheme in India is *Chiranjeevi Yojana* (CY), or “scheme for long life.” This state-level public-private partnership is administered and currently funded by the Gujarat government, although initially supported by JSY funds. Implemented throughout Gujarat by 2007, the “voucher like” scheme provides tribal and BPL women with free delivery in an accredited private facility and reimbursement for transport. CY has both demand- and supply-side components, increasing women’s demand for services while providing incentives to private providers for treating eligible mothers. CY was developed because three-quarters of Gujarati obstetricians worked in private health facilities, with large numbers in rural areas [5–7].

A growing number of studies have examined the impact of JSY and CY in increasing women’s use of maternity services [5–11]. However, many have focused on low performing states, limited geographic regions, and the processes involved in implementing JSY [3, 5, 8]. Thus, researchers have called for studies of high performing states that take different approaches to demand-side financing schemes, such as Gujarat and Tamil Nadu. Tamil Nadu’s introduction of JSY followed a period in the 1990s when its government increased the availability of round-the-clock obstetrical and newborn care through strategically located primary health facilities. Tamil Nadu uses federal funds to implement JSY, providing a cash incentive to BPL mothers who deliver in public health facilities. The state also offers women a small amount of cash for home births under another scheme to cover nutrition and lost wages [12]. In contrast, Gujarat has relied heavily on the CY scheme to provide poor, rural women with private maternity health services [6, 7], although eligible Gujarati women may also elect to deliver in government facilities under JSY.

Neither JSY nor CY incentivized antenatal care (ANC) in 2007–08 when the DLHS-3 was carried out. Both schemes provide small incentives to Accredited Social Health Activists (ASHAs) or health workers to accompany women to deliver in a health facility. Cesarean-sections (C-sections) are handled differently by the two schemes. Tamil Nadu’s JSY scheme provides obstetricians and anesthesiologists with an additional payment to perform C-sections in public facilities, whereas Gujarat’s CY scheme makes a flat rate payment to private physicians for 100 deliveries to cover normal and complicated births, thus removing the incentive to conduct surgical procedures [5–7].

Despite different financing mechanisms, JSY and CY both aim to reduce financial barriers to institutional deliveries among poor women. Other health system factors are also likely to influence women’s use of maternal health services, particularly in poor, rural populations [13–15]. One such health system factor is the availability of a primary health center that provides 24-hour basic emergency obstetric care [16]. Geographic access to a health facility may also impact service use because 40 % of Indian villages lack all-weather roads, 2.7 million kilometers of rural roads are in poor condition, and 33 % of Indian villages are often cut off from major services by monsoon flooding [17, 18]. Socio-demographic and obstetrical factors have likewise been found to influence maternal health services utilization, including women’s education, husbands’ education, women’s age at first birth, and parity [19, 20]. Given multiple factors that may influence use of maternity services, including financing schemes, this study sought to examine the role of JSY, CY and selected health system, socio-demographic, and obstetric factors in predicting use of adequate ANC, institutional delivery, and C-section among poor, rural women in Gujarat and Tamil Nadu.

## Methods

### Study setting and sample

This cross-sectional study utilized India’s District Level Household Survey (DLHS)-3 Ever-married Women and Village datasets [21]. The analytic sample included all ever-married women, ages 15–49, in the lowest two wealth quintiles (1st and 2nd) in rural areas of Gujarat and Tamil Nadu, who gave birth between January 2006 and December 2007. The total analytic sample of 2185 included 1584 Gujarati and 601Tamil women.

### Data collection

DLHS-3 (2007–08) data were collected by the International Institute for Population Sciences (IIPS), Mumbai, on behalf of the Indian Government’s Ministry of Health and Family Welfare [21]. DLHS-3 was the third in a series of district-level, national surveys in India that provided estimates of important indicators of reproductive, maternal, and child health; it is the latest national-level demographic survey that includes maternal health indicators. About 644,000 ever-married Indian women were surveyed in DLHS-3. Details concerning data collection, weights, and handling of missing data are described on the DLHS-3 website [22].

DLHS-3 definitions were adopted for all study variables. For dependent variables, adequate ANC was defined as having an ANC visit in the first trimester, at least three total antenatal visits, and at least 100 iron folic acid tablets taken during the last pregnancy, as reported by women participants. Institutional delivery was defined as delivery in a public institution (government hospital, dispensary, urban health centre/post/family welfare centre, community health centre/rural hospital, PHC, sub centre, and Ayurveda, Yoga, Unani, Siddha, and Homeopathy (AYUSH) hospital/clinic) or in a private hospital/clinic or private AYUSH hospital/clinic. C-section was defined as a delivery by this operation.

Independent variables included health financing scheme, health system, socio-demographic, and obstetric variables. JSY/CY assistance was defined as a woman’s report of receiving financial assistance for delivery care under either scheme. Health system variables were the village leader’s report of women having access to a primary health centre (PHC) with 24–7 services in the area (yes or no) and village access to a health facility via an all-weather road (yes or no). Socio-demographic variables included the woman’s education and her husband’s education, defined as each spouse’s total years of schooling. Obstetrical variables included age of the woman’s first birth (in years) and parity (primipara, multipara, grand multipara). The control variables of religion (Hindu or non-Hindu) and caste (scheduled caste or non-scheduled caste) were defined as the religion or caste of the household head.

### Ethical standards

Following the DLHS-3 protocol, informed consent was obtained from all participants before their participation. All identifiable information was removed from the dataset. The de-identified version of the DLHS-3 is available in the public domain [21, 22]. Ethical clearance was also obtained from the Institutional Research Board at the University of Maryland, where the data analyses were conducted.

### Statistical analyses

Survey data were analyzed using primary sampling units and state weights for ever-married women and households, determined by DLHS-3. Descriptive statistics summarized characteristics of the sample and women’s use of maternal health services in Gujarat and Tamil Nadu. Comparisons were made of these variables in the two states using Chi squares or a *t*-test (age of first birth). Relationships among all independent and dependent variables were examined using binary logistic regression. All independent variables were at least moderately (*p* < 0.10) correlated with one or more measures of maternity services utilization in each state. Multicollinearity was also assessed; all independent variables that remained in the final models had acceptable tolerance values. Multiple logistic regression analyses were then used to determine the degree to which independent variables predicted adequate ANC, institutional delivery, and C-section, controlling for religion and caste. Religion and caste were controlled because the literature indicates that both are predictors of Indian women’s use of maternal health services [19, 23]. Three separate regression analyses were carried out in each state, one for each dependent variable. Strength of association between study variables was estimated by calculating adjusted odds ratios (AORs) with 95 % confidence intervals (CIs); a *p* value of .05 or below was considered statistically significant. Hosmer-Lemeshow goodness of fit tests revealed that models for all dependent variables were a good fit for data in both states (*p* > 0.05). A complete case analysis was conducted for each of the dependent variables since some data were differentially missing for adequate ANC in the two states. Missing data for adequate antenatal care were 3.1 % in Gujarat and 0.5 % in Tamil Nadu. Statistical analyses were carried out with IBM SPSS 19.

## Results

Results revealed significant differences between samples of poor, rural Gujarati and Tamil women who gave birth in calendar years 2006 and 2007 with respect to JSY/CY participation, health care system, socio-demographic, and obstetric variables, as well as use of maternity services. Table 1 presents descriptive data for mothers in the analytic sample, including 1584 Gujarati and 601Tamil women. During the birth years studied, Tamil women (28 %) were three times more likely than Gujarati women (9 %) to receive JSY/CY assistance for their most recent birth. Tamil women (74 %) reported greater availability of a primary health care center operating round-the-clock than Gujarati women (58 %), and Tamil women (97 %) had greater access to a health facility by an all-weather road than their Gujarati peers (80 %). Although both Tamil Nadu and Gujarat are high-performing states, Gujarati women (71 %) were twice as likely to be uneducated as Tamil women (32 %). Husbands in both states were more educated than their wives, but Gujarati husbands (40 %) were more likely to be uneducated than Tamil husbands (28 %). Tamil women’s mean age at first birth (21.0 years) was significantly higher than that of Gujarati women (19.5 years), and there were also significant differences in parity, with a higher percentage of primipara and multipara women in Tamil Nadu and a higher percentage of grand multipara women in Gujarat. The vast majority (96 %) of women in both states were Hindu, but more Tamil women (95 %) than Gujarati women (54 %) were in the scheduled caste.

**Table 1:** Descriptive statistics for women from Gujarat and Tamil Nadu

Independent variables	Gujart (*n* = 1584) weighted %	Tamil Nadu (*n* = 601) weighted %	*p*-Value
Financial assistance from JSY or CY	08.8 %	28.3 %	0.00**
Availability of PHC providing 24/7 services	58.3 %	73.5 %	0.00**
Access to a health facility by an all-weather road	80.0 %	96.8 %	0.00**
Woman’s education			
Uneducated	71.3 %	31.8 %	0.00**
Less than 5 years	9.9 %	10.1 %	
5-9 years	16.5 %	44.7 %	
10 or more years	02.4 %	13.4 %	
Husband’s education			
Uneducated	40.1 %	28.0 %	0.00**
Less than 5 years	12.9 %	10.6 %	
5-9 years	32.9 %	46.3 %	
10 or more years	14.1 %	15.1 %	
Woman’s age at first birth			
Mean	19.5 yrs	21.0 yrs	0.00**
Minimum	10.0 yrs	14.0 yrs	
Maximum	35.0 yrs	37.0 yrs	
Parity			
Primipara	22.1 %	36.4 %	0.00**
Multipara	42.1 %	52.6 %	
Grand multipara	35.8 %	11.0 %	
Control variables			
Religion			
Hindu	95.8 %	96.4 %	0.51
Non-Hindu	04.2 %	03.6 %	
Caste			
Scheduled caste	53.8 %	95.4 %	0.00**
Non-scheduled caste	46.2 %	04.6 %	
Outcome variables			
Receipt of adequate antenatal care^a^	7.5 %	24.4 %	0.00**
Institutional delivery	30.9 %	85.3 %	0.00**
Percentage of private facility deliveries	52.0 %	19.0 %	0.00**
Percentage of public facility deliveries	48.0 %	81.0 %	
Cesarean section delivery	03.6 %	14.8 %	0.00**

**p < 0.01

^a^Antenatal care data were missing for 50 Gujarati women and 3 Tamil women

Examination of women’s use of maternal health services in the two states revealed significant differences for all three outcome variables. Tamil women (24 %) were three times as likely as Gujarati women (8 %) to report adequate ANC. Tamil women (85 %) were almost three times more likely to have delivered their last child in a health care facility than Gujarati women (31 %); however, it is notable that 52 % of institutional deliveries in Gujarat were in a private facility as compared to 19 % in Tamil Nadu. C-section deliveries were significantly more prevalent among Tamil (15 %) than Gujarati (4 %) women.

Predictors of maternal health services utilization among women in the two states, based on multivariate analyses, are summarized in Table 2.

**Table 2:** Adjusted odds ratios and confidence intervals for use of maternal health services by Gujarati women and Tamil women^a^

Predictors	Adequate antenatal care				Institutional delivery				Cesarean section			
	Gujarat		Tamil Nadu		Gujarat		Tamil Nadu		Gujarat (n=1584)		Tamil Nadu	
	(n=1534)		(n=598)		(n=1584)		(n=601)				(n=601)	
	AOR	95 % CI	AOR	95 % CI	AOR	95 % CI	AOR	95 % CI	AOR	95 % CI	AOR	95 % CI
Financial assistance from JSY or CY	0.83	0.42-1.63	0.92	0.61-1.39	3.93***	2.68-5.77	1.60	0.87-2.93	1.11	0.48-2.54	1.66*	1.01-2.70
Availability of PHC providing 24/7 services	0.70	0.44-1.11	0.90	0.59-1.38	1.04	0.83-1.31	1.04	0.62-1.75	1.16	0.93-2.34	1.59	0.90-2.81
Access to a health facility by an all-weather road	1.02	0.49-2.11	NA^b^	NA	1.42*	1.06-1.90	3.42*	1.24-9.47	0.71	0.38-1.34	0.44	0.14-1.31
Woman’s education												
Uneducated	Ref		Ref		Ref		Ref		Ref		Ref	
Less than 5 years	1.25	0.63-2.50	0.93	0.47-1.84	0.85	0.58-1.25	1.18	0.43-1.67	0.80	0.30-2.11	2.44*	1.05-5.66
5-9 years	1.32	0.74-2.34	1.12	0.70-1.78	1.01	0.74-1.38	2.34***	1.32-4.15	0.99	0.48-2.02	1.57	0.78-3.18
10 or more years	2.78*	1.08-7.14	1.09	0.56-2.10	0.85	0.43-1.69	2.97*	1.08-8.21	0.86	0.17-4.45	1.99	0.83-4.79
Husband’s education												
Uneducated	Ref		Ref		Ref		Ref		Ref		Ref	
Less than 5 years	1.87	0.99-3.54	1.36	0.71-2.62	0.72	0.50-1.06	1.15	0.49-2.72	0.67	0.22-2.01	0.83	0.31-2.22
5-9 years	0.94	0.53-1.68	1.01	0.62-1.64	0.99	0.76-1.30	1.17	0.64-2.13	1.20	0.62-2.32	0.93	0.49-1.79
10 or more years	1.72	0.91-3.26	0.97	0.51-1.86	1.44*	1.03-2.02	1.07	0.45-2.55	1.69	0.77-3.71	1.85	0.84-4.10
Age at first birth	1.07	0.99-1.16	1.04	0.98-1.09	1.05*	1.01-1.09	1.08†	1.00-1.17	0.94	0.83-1.06	1.08*	1.01-1.16
Parity												
Primipara	Ref		Ref		Ref		Ref		Ref		Ref	
Multipara	1.18	0.69-2.01	0.79	0.53-1.18	0.54***	0.41-0.72	0.65	0.51-1.10	0.44***	0.24-0.81	0.58*	0.36-0.96
Grand multipara	0.70	0.37-1.33	0.77	0.39-1.54	0.54***	0.40-0.72	0.43*	0.19-0.97	0.23***	0.10-0.51	0.18*	0.04-0.80

*p < 0.05, **p < .01, ***p < 0.001

^a^All analyses controlled for religion and caste

^b^NA. Variable removed from the model due to multicollinearity

### Adequate ANC

Receipt of JSY/CY financial assistance, availability of a PHC with 24/7 services, and access to a health facility via an all-weather road failed to significantly predict adequate ANC in Gujarat or Tamil Nadu. None of the socio-demographic or obstetric variables were significant predictors of ANC in either state, except maternal education in Gujarat. The odds of a Gujarati woman receiving adequate ANC were 2.8 times higher among women who had 10 or more years of education compared to uneducated women; however, the association was not significant at other levels of education.

### Institutional delivery

Participation in CY/JSY significantly predicted institutional delivery in Gujarat. The odds of delivering in an accredited health facility were 3.9 times higher among Gujarati participants in CY/JSY compared to non-participants in the schemes. No such relationship was found in Tamil Nadu. The odds of having an institutional delivery were 1.4 times higher among Gujarati women and 3.4 times higher among Tamil Nadu women when they could access a health facility via an all weather road compared to their peers without such access. Although woman’s education did not predict institutional delivery in Gujarat, it was a significant predictor in Tamil Nadu. The odds of delivering in a health facility for Tamil women with 5–9 years of education were 2.3 times higher and with 10+ years of education were 3.0 times higher compared to uneducated women. In Gujarat, husband’s education was significantly related to his wife’s institutional delivery for husbands with 10 + years of education; the odds of institutional delivery for Gujarati women whose husbands had completed secondary or higher education were 1.4 times higher compared to women whose husbands were uneducated. Age at first birth significantly predicted institutional delivery in Gujarat; with each 1 year increase in a woman’s age at first birth, the odds of having an institutional delivery increased by 5 %. Parity significantly predicted institutional delivery in both states; the odds of Gujarati multipara and grand multipara women having institutional deliveries were 46 % lower and Tamil grand multipara women were 57 % lower compared to primipara women in their states.

### C-section delivery

Receipt of JSY significantly predicted C-section in Tamil Nadu. The odds of having a C-section delivery were 1.7 times higher among JSY participants compared to non-participants. There was no significant association between CY/JSY and C-section in Gujarat. Woman’s education significantly predicted C-section for Tamil women but not for Gujarati women. Specifically, the odds of a C-section delivery were 2.4 times higher for Tamil women with 1–4 years of education compared to uneducated women. Age of first birth predicted C-section in Tamil Nadu but not Gujarat. With each 1-year increase in a Tamil woman’s age at first birth, the odds of a C-section increased by 8 %. Parity predicted C-sections in both states; the odds of Gujarati multipara women having a C-section were 56 % lower and grand multipara woman 77 % lower compared to primipara women. The odds of Tamil multipara women delivering by C-section were 42 % lower and grand multipara women were 82 % lower compared to primipara women.

## Discussion

Our study extends existing research on India’s health financing schemes by examining their operation in two high-performing states, and by including consideration of selected health system, socio-demographic, and obstetric factors in predicting use of maternity health services. Of key interest was women’s use of JSY or CY to cover the cost of their institutional births. Relatively low percentages of poor, rural women were beneficiaries of the schemes, with Tamil women three times more likely to report receiving government assistance than Gujarati women. Tamil women were also more likely than their Gujarati peers to report adequate ANC or have a C-section birth. Several contextual factors likely contributed to these findings. Tamil Nadu launched JSY in 2005, building on its former Muthulakshmi Reddy Maternity Benefit Scheme established in 1989, while Gujarat’s CY scheme was not implemented statewide until January 2007 [6, 12, 24]. Moreover, Tamil Nadu has long been recognized as a national leader in maternal health policy and public health system administration [25]. Annual per capita public health care expenditures are higher for Tamil Nadu at 200 INR compared to approximately 166 INR for Gujarat [26]. Lower government spending increases out-of-pocket healthcare expenses for items such as transportation that contribute to the full cost of institutional deliveries [27, 28].

A major study objective was to examine financial, health system, socio-demographic, and obstetric factors that predicted poor, rural women’s use of maternity services in the targeted states. None of the factors under investigation predicted women’s adequate ANC in Tamil Nadu, and a high level of maternal education was the only significant predictor of adequate ANC in Gujarat. The general failure of JSY/CY to predict adequate ANC after adjusting for socio-demographic factors was not surprising given that ANC was not incentivized during the study; women received no payments or vouchers for ANC and providers received no scheme incentives to provide the service. ASHAs received a one-time payment for women’s institutional deliveries regardless of whether they scheduled any ANC visits for these same women [29]. Another study examining the impact of JSY throughout India also found the program to have little or no impact on antenatal care [30]. It has been argued that JSY and CY should link incentives to ANC, institutional delivery, and postnatal care, providing a continuum of care for mother and child [6, 29]. Currently, few studies have examined the impact of incentivizing ANC [31]; one Honduran study estimated that a conditional cash transfer incentive significantly increased ANC [32] and a Kenyan study found that provision of free bed nets at an ANC clinic greatly increased uptake of prenatal services [33]. Another study revealed no impact of the Mexican conditional cash transfer scheme on ANC, but this finding was attributed to very high baseline rates [34].

Receipt of CY/JSY significantly predicted institutional delivery among poor, rural women in Gujarat, with the odds of women delivering in a health facility almost four times higher among those participating in either scheme compared to non-participants. This finding is consistent with previous research linking CY and other financial schemes to increased institutional deliveries [3, 5–7, 10]. In contrast, a recent study that collected retrospective information from a statewide sample of Gujarati women who delivered since January 2005, as well as DLHS-3 data (2005–2007), concluded that CY has not increased women’s probability of having an institutional delivery [11]. The latter study included both urban and rural Gujarati women and the retrospective component made no attempt to identify households with BPL status. Moreover, it was limited by respondents’ recall of their ANC and deliveries over a 5-year period (versus the maximum 2-year look back of DLHS-3), suggesting the potential for recall bias. These findings suggest that CY may have a differential impact on particular segments of the Gujarat population, an issue deserving of future research. Notably, an evaluation of JSY throughout India found the scheme to be most effective in increasing institutional deliveries among poor, less educated, and ethnically marginalized women [35].

Receipt of JSY did not predict institutional delivery among Tamil women in the study, the vast majority (85 %) of whom delivered their last child in a health facility. Tamil Nadu’s practice of providing some financial assistance to pregnant women even prior to JSY, regardless of whether they delivered in a facility or at home, may have contributed to this finding. The differential payment of INR 500 between institutional and home delivery suggests a relatively weak incentive for those who preferred a home birth [12, 24].

Access to a health facility by an all-weather road significantly predicted institutional deliveries in both Indian states, supporting the importance of addressing this variable in initiatives aimed at motivating rural women to deliver in health facilities. All-weather road access directly affects whether a woman can reach a facility for delivery, and indirectly affects her decision to pursue a facility delivery in the first place. Contrary to expectations, availability of a PHC with 24–7 services did not predict institutional delivery in either state. A number of supply-side constraints linked to PHCs, including unreliable staffing, supply shortages, and problematic referral transport for emergency complications, especially at centers below the district hospital level, may have diminished women’s motivation to deliver in some facilities [36–38].

Findings further revealed that JSY participation significantly predicted C-section deliveries in Tamil Nadu, but not in Gujarat. Almost 15 % of poor, rural Tamil women reported a C-section for their last birth, compared to less than 4 % of their Gujarati peers. The significant role of the financing scheme in predicting C-section deliveries in Tamil Nadu may be influenced by the state’s availability of health facilities providing comprehensive emergency care and JSY’s additional payment to obstetricians and anesthesiologists for C-section deliveries [12]. The CY scheme factors in an estimated proportion of C-sections in the flat rate payment to obstetricians for delivery care, and thus, does not incentivize surgical intervention. Notably, the Gujarat C-section rate (3.6 %) for women in this study was below WHO’s optimal range of 5-15 % for all births in a country and below India’s rural C-section rate of 6 % [39]. Thus, the risk with CY may be that there is little incentive for private obstetricians to accept women with complications as patients [5]. One recent study found that Gujarat’s C-section rate had increased to 6 % for CY beneficiaries between 2006 and 2010, but these data were not disaggregated for urban and rural, poor women [40]. The researchers noted that private obstetricians tended to be concentrated in Gujarat’s cities and small towns, and thus rural women were likely to experience greater barriers in accessing comprehensive emergency obstetric care.

Differential patterns were also found for socio-demographic predictors of women’s use of health services in the two states. For example, woman’s education was a significant predictor of institutional delivery and C-section in Tamil Nadu, while higher levels of husband’s education predicted institutional delivery in Gujarat. Previous research has found woman’s education to be a consistent predictor of institutional delivery in rural India [20]. Current findings further suggest that more educated husbands in Gujarat also understood the potential benefits of institutional deliveries. With respect to obstetric factors, age at first birth significantly predicted institutional delivery in Gujarat and C-section in Tamil Nadu; in both cases, older women were more likely to deliver in a health facility or obtain a C-section than their younger peers, suggesting that adolescent women may be underserved. Finally, parity significantly predicted institutional delivery and C-section in both states, with multiparous women less likely to utilize these maternity services. Possible contributors to this finding include JSY’s policy of incentivizing only the first two live births [4, 5], limited financial resources of larger families, greater confidence of multiparous women with a history of uncomplicated births, and/or women’s possible dissatisfaction with health facilities.

### Limitations

Several limitations of our study must be noted. The DLHS-3 collected data on births in 2006 and 2007, with most births occurring in 2007 [21]. Given the frequent time lag between the launch of new policies and effective implementation at the grass roots level, findings may not reflect the current influence of the schemes. Another limitation is the possibility of recall bias, both on the part of women who were asked to remember details of their antenatal and delivery care, and village heads who reported on access to a PHC and an all-weather road. However, the recall period in this study was relatively short (two years) and DLHS-3 field interviewers were well trained to collect retrospective data.

Additionally, the study’s cross-sectional design precludes concluding causality from observed relationships. Our investigation of poor, rural women in two high-performing states likewise prevents generalizing the findings to all Gujarati or Tamil women, or to all low-income, rural women in India. Future research, including studies with longitudinal designs in high- and low-performing states, are needed to assess the impact of financial schemes, health system, and other social factors on Indian women’s use of maternal health services, including ANC. Future research might also examine how the relationship between financial assistance from JSY or CY and women’s use of institutional delivery is modified by various health system factors such as referral transport and availability of skilled human resources, and various social factors such as women’s autonomy.

### Policy and program implications

Based on our findings, we recommend that policy makers, program planners, and practitioners consider the following strategies for improving access, use, and quality of maternal health services for poor, rural women in the target states and throughout India.

First, efforts should be made to increase the use of demand-side maternal health financing schemes in the targeted states through health information, education, and communication campaigns aimed at the literate and non-literate, rural poor. ASHAs and community health workers play key roles in enrolling women in the schemes early in their pregnancies and require training. Given the link between incentives and service use, Tamil Nadu might consider eliminating the cash payment for home births to provide a stronger incentive for institutional delivery. States should also consider assigning part of the JSY payment for ANC, with the goal of educating women about fetal growth and nutrition, detecting high risk pregnancies, and developing advance plans for delivery/emergency care.

Second, states should augment promotion of demand-side financing schemes with improvements in the quality of maternal health services for poor, rural women. Enhancements in facility infrastructure, human resources, transport systems, referral coordination, supply chain systems and respectful evidence-based care are likely to improve health outcomes, as well as increase patient satisfaction and future service use.

Third, states should increase geographic access to health care facilities by supporting government investment in rural road infrastructure, including construction of all-weather roads. Such efforts may reduce both direct transportation costs and opportunity costs of seeking care in facilities that are far from rural women’s homes.

Fourth, support for increasing the education and literacy levels of poor, rural women may enhance their ability to make wise decisions about appropriate reproductive care. Health educational campaigns targeting women, husbands, and key household members may increase women’s access to valuable maternity services.

Finally, researchers should use both qualitative and quantitative methods to explore poor, rural women’s experiences with existing financing schemes, their opinions about the quality of health facilities, and their unmet obstetric needs. Similarly, health impact evaluations should collect qualitative data from health care professionals and managers about contextual factors that influence provision and use of maternity services, with the goal of strengthening understanding of quantitative outcomes.

## Conclusion

Differential patterns of findings for the Indian states of Gujarat and Tamil Nadu underscore the complexity of designing interventions to enhance poor, rural women’s use of maternity services. Findings suggest the need for both states to increase women’s participation in health financing schemes that incentivize institutional delivery, as well as improve women’s access to ANC. States should conduct careful contextual analyses and leverage existing resources to develop policies and programs that enhance the quality, use, and equity of maternal health services. Additional evaluation of JSY, CY, health system and social factors may strengthen interventions aimed at reducing India’s maternal mortality ratio and influence maternal health schemes for poor women throughout the world.
